# The Validity of Visual Communication Design of Online Advertisement Based on Interactivity

**DOI:** 10.1155/2022/2485809

**Published:** 2022-07-08

**Authors:** Ruizhi Zu

**Affiliations:** Taiyuan Normal University, Taiyuan 030619, China

## Abstract

Today's society is a society of Internet information, especially the rapid development of new media that is the most distinctive project today. New media has changed the way traditional media are disseminated, and the advertising industry has made new developments with the rapid development of new media, especially with the rise of online advertising, making the dissemination of advertising more influential. In modern advertising information, online advertising has become very influential. This study aims at how to achieve user-matching push and set the right price is the two major problems of Internet advertising push, through the analysis of the emergence and development of network advertising and visual elements in network advertising, starting from the problems appearing in modern online advertisements, and studying the visual communication design in online advertisements. In this paper, a neural network model and a fuzzy comprehensive evaluation decision model are designed; so as to make our online advertisements rich in national characteristics and really cause online users' active clicks. The experimental results show that the proposed method achieves good results and verifies the effectiveness of the model.

## 1. Introduction

Visual communication design is the active behavior designed to propagate specific things through a visual form, and it is a design activity that uses stylistic means and various visual media to communicate information, including graphics, colors, images, and other expression methods to produce certain artistic effects. Advertising design belongs to the category of visual communication design. In the artistic creation process of advertising design, information is conveyed to the public mainly through the aesthetically pleasing organization of graphics, text, color, and other elements, which consist of two parts: basic visual elements and design principles. The basic visual elements include lines, colors, and space, and the design principles include layout, contrast, metaphor, exaggeration, and other ways. As a visual medium in the new era, interactive network advertising design is both a continuation of traditional advertising design and has its characteristics and advantages of network communication. When designing in practice, designers should select basic visual elements and follow corresponding design principles based on the characteristics of network advertising design, advertisers' purposes, and consumers' needs.

With the continuous development of science and technology and the maturity of network technology, the network has become a new way of information dissemination, and become the fifth largest media in the world—network media. In the twenty-first century, the world has entered the digital information era. The use of network media to transmit information has become a fashion, breaking through the limitations of traditional information transmission methods, not only breaking the geographical and cultural barriers but also increasing the speed, efficiency, and scope of communication that has been qualitatively improved. Various traditional media have gradually appeared online, such as online newspapers, online magazines, and online TV. At the same time, new forms of dating have also emerged, such as domestic dating sites like Jenai.com and CenturyJiaYuan.com. The foreign Twitter and Facebook are the pioneer representatives of dating sites. These new modern websites and media networks have gradually promoted the development of online advertising. The development of online advertising is also accompanied by the mushrooming of various online media.

Online advertising in China has shown great market potential, and online advertising has become the preferred form of advertising operation for various advertisers. When you turn on the computer, there are many kinds of advertisements in front of the browsing users, but how to make the Internet users click on these online advertisements actively has become a more concerned issue for the designers of online advertisements. Although the development of modern online advertising is inseparable from the development of online information, it is even more inseparable from the development of visual communication design. Online newspapers, online magazines, and online TV exist in the form of independent web pages, but online advertisements are part of these web pages, and the click-through rate largely depends on the visual communication design of online advertisements. Therefore, online advertising must follow some characteristics of visual communication design, but it is different from traditional visual communication design and has its own unique design guidelines. The design criteria of network advertising include advertising design to be creative, variable forms, advertising bar, and content is clear and concise and appealing, not only to provide visitors with your products or services but also to provide them with other valuable information. Only by fully understanding the visual communication design can we make the designed advertisement more suitable for the communication mode of this new media of network. Only then can online advertising gradually develop into a new modern medium with artistic, aesthetic, and great commercial potential.

The development of online advertising in China is later than that of online advertising in foreign developed countries, and its research is also relatively weak. In 2001, Jin Lin and Zhao Haibin edited “Network Advertising Design,” which mainly focuses on the form, creativity, and design of network advertising. In 2009, [1] edited “Network Advertising Design,” which contains an overview of network advertising, the design theory of network advertising, as well as the production of network advertising design examples and the appreciation of network advertising creativity. In 2010, [2] edited “Network Advertising Design,” which includes the planning of advertising and network advertising and the application of various software in network advertising, as well as the release of network advertising and the management and monitoring of network advertising.

The book *Branding in the Network Era* edited by US Sage focuses on the importance of visual expression in brand building; “Practical Course on Advertising Design in the U.S.” written by U.S. Robin Landa integrates advertising concepts and design strategies, and talks about the design and production methods of one of the advertisements, network advertising; the book *Changing Media Form a Understanding the New Media* edited by U.S. Roger Fiedler mainly introduces the new medium of online media. The book *Communication Media in the Information Age* by Joseph Straubach and Robert LaRose, USA, also focuses on the Internet as a medium from the perspective of media morphology [3]. *Marketing Management* edited by Philip Kotler, a professor at Northwestern University, USA, systematically discusses in detail the use and impact of the Internet and marketing management and related knowledge. *Branding on the Web*, edited by Kevin Lai, USA, discusses in detail how businesses can advertise on websites and develop their brands for maximum commercial return [4].

## 2. State of the Art

With the diversification of the network and the interactive characteristics of online advertising, the main visual communication technologies include data mining, natural language processing, etc., and there have been many mature studies in advertising click-through rate prediction. Logistic regression (LR) models directly model the probability of whether a user clicks on an advertisement, which is efficient and robust to small noise in the data through the use of regularization, and is the most commonly used online advertising click-through prediction model in industry. Jiang et al. [5] used logistic regression and Multiple Additive Regression Tress (MART) model for training respectively, and the experimental results showed that logistic regression outperformed MART [6]. This method uses L21-mixed regularization to reduce the computation time of the parameters as well as the logarithmic loss of the model. However, the performance of the logistic regression model is very dependent on the features involved in training, and its performance is limited in sparse data. An appropriate automatic feature extraction model is an effective tool to improve the prediction performance of logistic regression models.

About improving the performance of these predictions, Wu et al. [7] proposed an advertising click-through rate prediction model based on the Least Absolute Shrinkage and Selection Operator (LASSO) variable selection method, which effectively overcomes the problem of high dimensionality of advertising data by eliminating some features that are not relevant to prediction, and moderates the overfitting of prediction results to a certain extent. However, the LASSO variable selection method simply extracts those features that are more important to the prediction from the original features, which are not valid for click-through rate predictions. Wong and Kim [8] introduced the Coupled Group LASSO (CGL) model used by Ali, which uses Group LASSO to regularize user features and ad features, avoiding the introduction of too large a matrix and reducing the parameters of the model. However, the LASSO variable selection method simply extracts those features from the original features that are more important for prediction, and the selected features are not effective for click-through rate prediction. In order to achieve automatic combination of features, Peter and Funta [9] proposed the use of gradient boosted decision tree (GBDT) model to automatically combine and transform the features in the original features, and experimented with logistic regression model on the transformed features. Nikolaos et al. [10] used GBDT to extract features from advertisements in Spark Big Data distributed platform, and the experimental results proved that GBDT model also helps to improve the accuracy and performance of the prediction model in distributed environment. GBDT processes each feature individually during feature processing and does not learn the interaction relationships in the features. The LASSO variable selection method and GBDT are two feature extraction methods that are easy to implement, but they often fail to achieve good prediction results because they ignore the interrelationship between features.

Deep learning is now the most powerful data processing tool in computer vision, becoming the technology of choice for tasks such as classification, segmentation, and detection. Deep learning can automatically explore local dependencies between data and can build dense representations between features, enabling neural networks to extract higher-order features from raw data, and this ability to efficiently learn higher-order implicit information has also been applied to CTR prediction. Aghdam et al. [11] proposed a feature dimensionality reduction method based on tensor decomposition to mine the highly nonlinear correlations existing between features using a stacked self-coding network algorithm. Hikmat Fauzi et al. [12] combined K-means clustering algorithm and tensor decomposition to reduce the dimensionality of high-dimensional advertising features, and then used the reduced-dimensional data to predict the click-through rate of online advertising using Deep Belief Network (DBN) to verify the effectiveness of the model [13]. Wide models refer to traditional models that require manual feature selection, such as logistic regression models. Deep models refer to models that can automatically perform feature transformation, such as factorization machine models. Wide and Deep models are obtained by combining wide models with deep models.

## 3. Methodology

### 3.1. Principles of Modern Online Advertising Visual Communication Design

**Figure 1:**
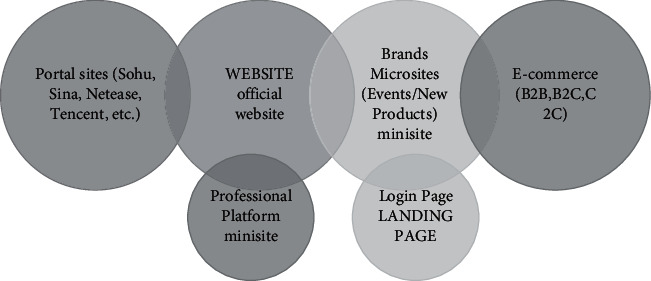
Interactive online advertising publishing platform.

1. Aesthetic principles of visual communication design for modern online advertising visual communication design requires the use of constant changes in images and the use of perspective and exaggeration and other methods. Let the creative aesthetics of the design be effectively enhanced through reasonable arrangement of the internal text of the advertisement. At the same time, the composition of the advertisement is constantly improved and only then can the beauty of color be reflected in the whole advertisement. Let the ad's viewability be enhanced. Make it more and more relevant to the public aesthetics [14].The realization of the communication effect of network advertising is mainly completed through the visual experience of network users. The effect of network advertising communication largely depends on the attraction of the images, text, color, and web layout of network advertising to the audience. Some forms and content are unique, and unique advertising works are easy to attract the attention of network users, so as to achieve the expected communication effect of network advertising.
2. Humanization principle of visual communication design of modern network advertisement. The main goal of advertising is to make the content and products introduced in the advertisement acceptable to the many viewers who see it, and the visual communication design is to make the main purpose of the advertisement better and more effectively realized. Visual communication design must always adhere to the principle of humanization so that the needs of the audience can be better met. Let the color and composition of the advertisement can be widely recognized by many viewers. In addition, in the process of visual communication, design should also respect the individual. Thus, viewers of different ages and cultural backgrounds can get the maximum satisfaction of their perceived needs. [15].
3. The innovation principle of modern network advertising visual communication design. In the current social background, everything needs to be constantly innovated in order not to be discarded by the times. The same is true for visual communication design. Designers should make a brave breakthrough to the traditional design model. Let the novelty and creativity be shown in the work. The style of advertising works can be more unique and different. The graphics and colors should be chosen with the purpose of the advertisement; thus, the advertising work can have a stronger influence. It is also possible to strengthen the expressive power of the advertising work [16].
4. The comprehensive principle of modern online advertising visual communication design. Visual communication design should reasonably use an integrated approach in the process of achieving design goals. In the design process, the correlation between each design element should be fully considered. We should be good at capturing the connection between the visual elements. The basic design rules should be effectively mastered, and the design methods should be flexibly applied to skillfully integrate the design methods to bring out the integrated role of many design solutions [17], and then effectively improve the quality and effect of the design (as shown in Figure 1).

### 3.2. Introduction of BP Neural Network Model

Due to the variety of types of Internet advertising and user characteristics, in order to improve the design quality and design effect of modern network advertising visual communication design, a BP neural network model with multiple inputs and outputs is chosen in this paper. The data collected from the questionnaire is preprocessed to build a BP neural network; the matching push is done according to the training model [18].

**Figure 2:**
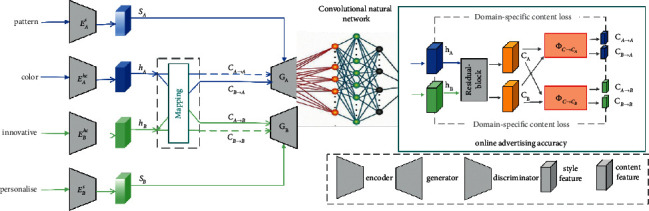
Construction of precision advertising model based on neural network model.

BP neural network is a multilayer feed-forward neural network, and the main feature of this network is forward transmission of signals and backward propagation of errors. In forward transmission, the input signal *y* is processed layer by layer from the input layer through the implicit layer to the output layer. The state of neurons in each layer only affects the state of neurons in the next layer. If the output layer does not get the desired output, it is transferred to back propagation, and the network weights and thresholds are adjusted according to the prediction error, so that the predicted output of the BP neural network keeps approaching the desired output. Since there are various types of Internet advertisements and various characteristics of Internet users, the characteristics of multiple inputs and multiple outputs and the BP neural network model have a good fit, so this paper decides to use the BP neural network model to solve problem one. One point to note is that although this paper preprocesses the collected data and excludes the wrong data as much as possible, the output results still have some deviation [19]. To solve the bias of the experimental results, we simulated this using a three-layer BP neural network model to facilitate the understanding of the implementation of the three-layer BP neural network model, and the following schematic diagram (Figure 2) can be borrowed to assist in the understanding.

### 3.3. BP Neural Network Modeling

In order to improve the practicality and operability of the model, a three-layer structure of BP neural network model is selected in this paper, and the specific meaning of each layer is

Input layer: The characteristics of TV channel users used this as the input layer, that is, age, gender, hobbies, profession, income, educational background, time spent watching TV, and type of products purchased by Chongqing TV users. Obviously, the input layer has eight nodes; denoted by, *x*_1_, *x*_2_,…, *x*_*s*_, respectively.

Output layer: The output of BP neural network is the type of video advertisement pushed by the TV station, including beauty, medicine, food, home life, school supplies, plants and flowers, etc. In this problem, six types of advertisements are selected as the output layer. In this paper, we use *y*_1_, *y*_2_, *y*_3_. to represent [20], respectively.

In the BP neural network model, the activation function plays an important role in the output results, and in order to make the output results more meaningful, the s-type function is chosen:

(1) $$\begin{array}{c} Y=\frac{1}{\left(1+e^{\left(-X\right)}\right)}. \end{array}$$

As the activation function of the hidden layer, the hyperbolic tangent function is used as the activation function of the output layer in order to speed up the learning rate of the neural network and reduce the number of iterations:

(2) $$\begin{array}{c} \tanh\left(x\right)=\frac{\left(e^{x}-e\left(-x\right)\right)}{\left(e^{x}+e^{\left(-x\right)}\right)}, \end{array}$$

where the actual output of the implicit layer neuron is

(3) $$\begin{array}{c} y_{j}\left(p\right)=\text{sigmoid}\left[\sum_{i=1}^{8}\left(x_{i}\left(p\right)w_{ij}\left(p\right)-\theta_{j}\right)\right]. \end{array}$$

Similarly, the actual output of the output layer neurons can be obtained as follows:

(4) $$\begin{array}{c} y_{k}\left(p\right)=\text{sigmoid}\left[\sum_{j=1}^{7}\left(x_{jk}\left(p\right)w_{jk}\left(p\right)-\theta_{k}\right)\right]. \end{array}$$

In summary, a three-layer BP neural network model is developed in this paper.

### 3.4. Fuzzy Integrated Evaluation Model

Since the pricing of TV commercials takes into account several factors, and the degree of influence of some factors on the price is difficult to measure precisely in quantitative terms, that is, the advertising price influence system has the characteristic of “fuzziness,” a fuzzy comprehensive evaluation decision model is adopted in this paper. Fuzzy comprehensive evaluation model refers to the map of the sum that is not absolute, and it is based on the importance of the ground, is the use of fuzzy comprehensive decision in fuzzy mathematics, and then can be judged under the influence of multiple factors. The three main influencing factors, such as program ratings, viewers' desire to buy, and purchasing power, were studied and matched with the data from Chongqing TV's advertising price list over the years to estimate a reasonable base price for each time slot [21].

In this paper, we first make a set of all the factors that may affect the pricing of Chongqing TV ads, denoted as follows:

(5) $$\begin{array}{c} U=\left\{u_{1},u_{2},u_{3},u_{4}\right\}. \end{array}$$

In this question, the following four influencing factors are selected: *u*_1_: the purchase desire of TV users for advertising products in this period; *u*_2_: the ratings of Chongqing TV in this period; *u*_3_: the pricing of advertisements in this period by other TV stations; and *u*_4_: the number of buyers bidding for advertisements in the same period.

(1) Suppose there are five evaluation levels, noted as follows:
  (6) $$\begin{array}{c} V=\left\{v_{1},v_{2},v_{3},v_{4},v_{5}\right\}, \end{array}$$
Where *v*_1_, *v*_2_, *v*_3_, *v*_4_, *v*_5_ represent this period of advertising bidding base price can be bid “very high, high, normal, low, very low.”
(2) For each of the four influencing factors, an evaluation set consisting of five rubrics is created, noted as follows:
  (7) $$\begin{array}{c} V_{i}=\left\{v_{i}{\;}_{1},v_{i2},v_{i3},v_{i4},v_{i5}\right\}. \end{array}$$

Specifically, the desire to buy the advertised product is “very high, high, normal, low, very low.” The number of times of watching TV in this period is “many, many, normal, little, very little.” The pricing of advertisements in this period by other TV stations is “high, high, normal, low, very low.” The number of buyers bidding for advertisements in this period is “many, many, normal, little, very little” and “Very high, high, normal, low, very low.” The number of buyers bidding for ads in the same time period is “many, many, average, few, very few.”

The evaluation matrix *R* is shown in equation.

(8) $$\begin{array}{c} R=\left(\begin{array}{c} R_{1}\\ R_{2}\\ R_{3}\\ R_{4} \end{array}\right)=\left(\begin{array}{ccccc} r_{11} & r_{12} & r_{13} & r_{14} & r_{15}\\ r_{21} & r_{22} & r_{23} & r_{24} & r_{25}\\ r_{31} & r_{32} & r_{33} & r_{34} & r_{35}\\ r_{41} & r_{42} & r_{43} & r_{44} & r_{45} \end{array}\right). \end{array}$$

Weights are relative values indicating the importance of factors and can be obtained by collecting publicly available statistics, questionnaires, and expert scoring methods to obtain the weight vector of evaluation factors *W* [22]:

(9) $$\begin{array}{c} W=\left\{w_{1},w_{2},w_{3},w_{4}\right\}. \end{array}$$

Synthesize *W* with *R* using the appropriate operator:

(10) $$\begin{array}{c} B=W\circ R. \end{array}$$

Then there is a combined evaluation result as shown below:

(11) $$\begin{array}{c} B=\left(w_{1},w_{2},w_{3},w_{4}\right){}^{\circ}\left(\begin{array}{ccccc} r_{11} & r_{12} & r_{13} & r_{14} & r_{15}\\ r_{21} & r_{22} & r_{23} & r_{24} & r_{25}\\ r_{31} & r_{32} & r_{33} & r_{34} & r_{35}\\ r_{41} & r_{42} & r_{43} & r_{44} & r_{45} \end{array}\right)=\left(b_{1},b_{2},b_{3},b_{4},b_{5}\right), \end{array}$$

where *b*_*i*_ is obtained from the *i*-th column operation of *W* and *R*. It indicates the affiliation of the settable advertising bidding reserve price high or low to the *v*_*i*_-level fuzzy level.

## 4. Result Analysis and Discussion

### 4.1. Study of Model Evaluation Indicators

**Figure 3:**
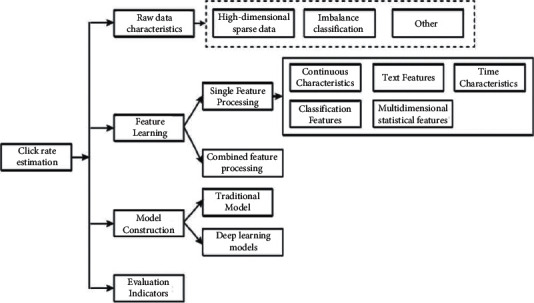
Framework of online advertising click-through rate estimation overview.

Click-Through Rate (CTR) and Conversion Rate (CVR) are important metrics to measure the effectiveness of ad delivery. Among them, the advertising click-through rate refers to the probability of being clicked by the user after the advertisement is officially released. Conversion rate refers to the probability that the user will actually consume after clicking on the ad. Relative to the ad click-through rate, the conversion rate directly affects the advertiser's business goals and economic benefits. The huge amount of user data gives the possibility of precise placement of online advertisements. With further research on the conversion of advertising click-through rate in industry and academia, it is necessary to systematically sort out the related literature, summarize the current status of research on the conversion of advertising click-through rate at home and abroad through analysis, and explore the research outlook for improving the return on investment (ROI) in advertising placement. The literature review on advertising click-through rate estimation is currently available, including the study by Wendy Ji et al. The author has used “CTR estimation” and “feature engineering + advertising” as topics in China Knowledge Network and “CTR” in the arXiv e-prints. “The search results were filtered, and the research content in this field can be roughly divided into four categories: original data characteristics and solutions, feature learning of click-through rate estimation, construction of click-through rate estimation models, and selection of evaluation indexes” (see Figure 3).

To investigate the impact of real-time user feedback on CTR, that is, whether the current click rate of users in the same session is highly correlated with their previous behavior, four user real-time feedback factors were analyzed in relation to real-time CTR, as shown in Table 1.

The main evaluation metrics used in the literature are: mean absolute error means absolute error (MAE), *F*-measure, AUC value, Log-loss, PR curve, ROC curve, relative information gain (RIG), accuracy, and recall.  Table 2 shows the use of each metric.

The model is evaluated by using AUC score, which is defined as the area enclosed by the axis under the ROC curve, and the horizontal and vertical coordinates of the ROC curve are the false positive rate and the true rate, respectively. The AUC score can be interpreted as the probability that a positive sample ranks ahead of a negative sample, and the AUC value is 1 when the prediction result is completely correct, so a larger AUC value indicates a more accurate classification result.  Table 3 shows the confusion matrix.

**Figure 4:**
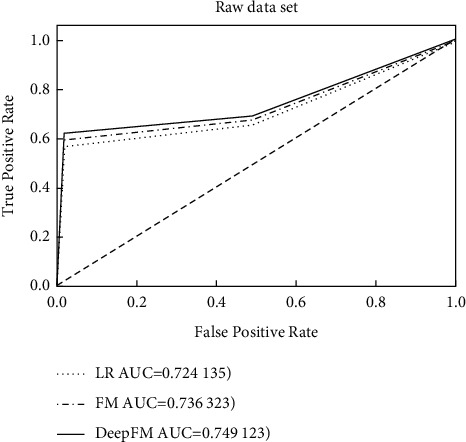
ROC curves of the original data set.

In this experiment, the three models were trained several times, and the final results were taken as the average AUC values of multiple experiments, and the experimental results are shown in Table 4 and Figure 4.

From the results of Table 4, we can see that due to the introduction of the second-order combinatorial feature, the AUC value of the FM model is 0.736323 and the AUC value of the LR model is 0.724135, which can be seen in the FM model, while the Deep-FM model has a higher AUC value than both the LR and FM models because of the inclusion of the deep neural network part to learn higher-order feature combinations. Analyzing Figure 4, we can see that the AUC value of Deep-FM is higher than both LR and FM because the positive and negative samples are unbalanced, so the front of the curve approximates a straight line *x* = 0, and the true rate of Deep-FM is higher than LR and FM at the back of the ROC curve. Combining the results in Table 4 and Figure 4, it can be shown that the Deep-FM model can effectively mine higher-order combinatorial features and has better recommendation effect than both LR and FM models.

**Figure 5:**
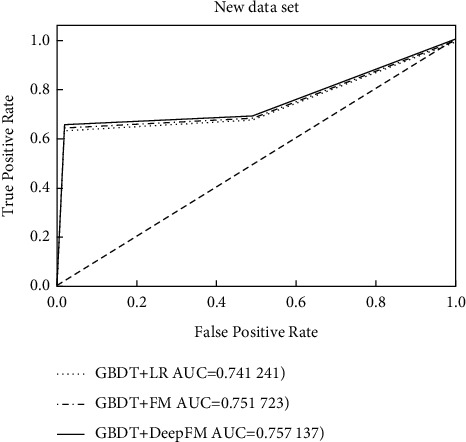
ROC curve for new data set.

In actual use, the logistic regression model as a linear model is more dependent on the manually extracted combinatorial features, so in this paper, based on the original features, we adopted the method in the literature and supplemented the dataset with new combinatorial features extracted by GBDT, and the final results are shown in Table 5 and Figure 5.

### 4.2. BP Neural Network Actual Measurement Results and Analysis

Since the data collected in this paper are from questionnaires, in order to improve the validity of the data, data cleaning, and data processing integration are needed for the collected data before data normalization or data dimensionality reduction can be performed.

Illegal data: In this paper, we found that the information filled in the questionnaires by the customers of Chongqing TV station was filled in arbitrarily. In terms of the questionnaire gender information, it is common sense to say that only men and women choose one of them, but this paper found that some questionnaire customers checked both of them. Therefore, these records need to be filtered and cleaned up, and this paper uses the deletion operation, that is, the class of illegal data, directly to delete the customer records. Because if the customer is not willing to provide the basic information on gender, it is difficult to expect the customer to provide effective real feedback on Chongqing TV, the results proved that the method is effective. For other illegitimate data, a similar method is used in this paper.

Missing data: As customers fill in the Chongqing TV station questionnaire, there are many nonhuman factors, such as no timely check, fill in time panic and other situations, resulting in some questions that are not filled in, resulting in the situation of null values. In order to make the data not empty, this paper uses the mean method, that is, the data of empty values are filled with the mean of that feature, thus increasing the reliability of the sample.

Data normalization: In this paper, the minimum–maximum normalization is used. BP neural network initialization: The weights and thresholds of the BP neural network model are randomly initialized.

The BP neural network is trained with training data, and the weights and thresholds of the network are adjusted according to the network prediction errors. In this paper, we build the model with user's age, gender, hobby, profession, income, educational background, TV viewing time, and product type purchased as input layer, six types of advertisements as output layer, and one implicit layer.

**Figure 6:**
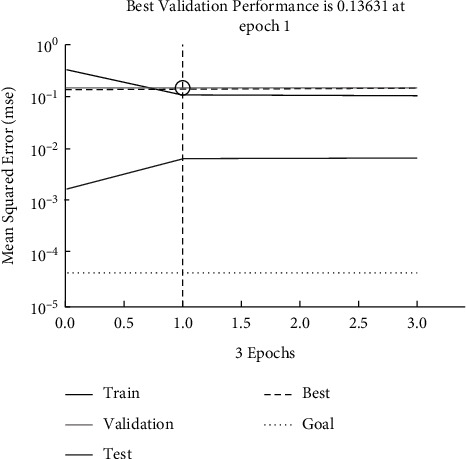
Variation of error squared during model training.

As can be seen from Figure 6, when the training sample is 200, the model takes 8,972 iterations to converge. After training the network, the learning mode of the network can be used to know what ads should be pushed to the user when the characteristics of the user are known.

**Figure 7:**
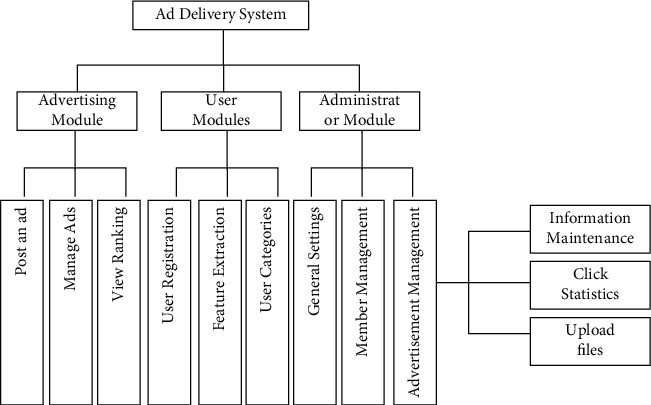
System framework diagram.

With the above analysis of user characteristics and the use of neural network technology to classify users, this paper further designs the framework of the precision advertising delivery system Figure 7.

## 5. Conclusion

The module to realize user classification using the neural network technology proposed in this paper is mainly for the user module, in which user registration information, and user behavior characteristics are extracted, and then the neural network technology is used to classify users, and the corresponding category of advertising information is placed for different categories of users, so as to achieve the purpose of accurate advertising delivery to users.

This paper only elaborates and studies the visual communication as a perspective. The success of online advertising should not only consider the smooth dissemination of advertising information but should also operate visual arrangement, and being able to attract the eye is the basis that all advertisements must have. Therefore, online advertising should not only meet the requirements of the audience, but it is also necessary to create a visual environment in a reasonable way so that the audience can actively accept the advertising information and even participate in the advertisement.

## Figures and Tables

**Table 1:** Relationship between real-time user feedback factors and real-time CTR.

Influence factor	Relationship with real-time CTR
Number of times the user clicks on the advertisement	Directly proportional
Number of times users ignore advertisements	Inversely proportional
Ad click interval	The interval between the occurrence time of more than 80% clicks and the last click is less than 1 minute.
	The longer the time interval from the last click, the greater the decline of users' real-time CTR.
Duration of user advertisement	The duration of advertising detention exceeds 30 seconds, and the user's real-time CTR shows a significant downward trend

**Table 2:** Statistics on the use of model evaluation indicators.

Evaluating indicator	Indicator description	Literature
MAE	Measure the distance between the estimated value and the real value	[22]
F-measure	The evaluation algorithm estimates the accuracy, which integrates the precision and recall in the field of information retrieval	[39]
AUC	The larger the AUC, the better the performance of the prediction model. AUC focuses on the ranking of advertising click-through rate estimation, so there is little change when the click-through rate increases by a certain proportion	References [11, 13, 16, 19] [30, 38, 40]
Logarithmic loss (logloss)	It is often used in binary classification problems to measure the inconsistency between the estimated value and the real value of the model. The lower the value, the better the effect of the evaluation model	[7, 21]
PR curve	That is, the precision recall curve. In the case of very few positive examples, the performance effect of PR curve is better than ROC curve	[40]
ROC curve	Based on the confusion matrix, it is mainly used to compare the estimated results with the real results	[40]
RIG	Relative information gain	[30]
Accuracy	Due to the serious imbalance of advertising data categories, it is inappropriate to use the accuracy index to measure the performance of the model	[22]
Recall	Measure the proportion of all positive examples that are estimated to be correct.	[22]

**Table 3:** Confusion matrix.

Forecast actual	Forecast	
	Positive	Negative
Positive	TP	FP
Negative	FN	TN

**Table 4:** Results of the original dataset.

Model	AUC
LR	0.724135
FM	0.736323
Deep-FM	0.749123

**Table 5:** Supplementary data set results.

Model	AUC
GBDT + LR	0.741241
GBDT + FM	0.751732
GBDT + Deep-FM	0.757137

## Data Availability

The labeled data set used to support the findings of this study is available from the corresponding author upon request.
